# CRU–Urea Mixtures Improve Maize Protein Yield and Nitrogen Use Efficiency in the Black Soil Region of Northeast China

**DOI:** 10.3390/plants15050675

**Published:** 2026-02-24

**Authors:** Lele Tian, Chunyan Yin, Liang Feng, Xiaorong Wu, Li Han, Jinhu Yang, Fang Luo, Ju Zhao, Lijun Li

**Affiliations:** 1 Inner Mongolia Academy of Agricultural & Animal Husbandry Sciences, Huhhot 010031, China; tianlele@emails.imau.edu.cn (L.T.); ycyliuhu@163.com (C.Y.); 13474703792@163.com (L.F.); 2 College of Agronomy, Inner Mongolia Agricultural University, Huhhot 010019, China; shendu2012126636@imau.edu.cn (X.W.); hanli@emails.imau.edu.cn (L.H.); yjh0019@emails.imau.edu.cn (J.Y.); 3 Arong Banner Agricultural Development Center, Hulunbuir 162750, China; 18848177303@163.com

**Keywords:** CRU–urea mixtures, synchronized nitrogen supply, nitrogen utilization efficiency, nitrate leaching, yield

## Abstract

Excessive nitrogen fertilizer application in the black soil region of Northeast China leads to nitrate leaching and gaseous nitrogen loss, posing environmental risks. This study aimed to evaluate the effectiveness of controlled-release urea (CRU) mixed with conventional urea in synchronizing nitrogen fertilizer supply with maize nitrogen requirements, improving nitrogen fertilizer use efficiency (NUE), and increasing economic benefits. A two-year field trial (2023–2024) tested six nitrogen fertilizer application strategies, all with a total nitrogen application rate of 168 kg N ha^−1^, including no nitrogen fertilizer application (CK), conventional fractionated urea application (C0), and four controlled-release urea–urea mixed application schemes, where CRU supplied 100%, 70%, 50%, and 30% of the total nitrogen (C100, C70, C50, and C30). The results showed that the C70 treatment had the highest maize grain yield and protein yield, at 12,502.92 kg ha^−1^ and 1567.65 kg ha^−1^, respectively, and NUE increased by 10.07% in 2024 compared to the C0 treatment. The C70 strategy also reduced nitrate concentrations in deeper soil layers, decreasing nitrogen loss by 29.04–31.21% compared to the C0 treatment. Furthermore, the C70 strategy yielded the highest net benefit, reaching $2817 ha^−1^. These results indicate that in black soil systems, a single basal application of C70 mixed fertilizer is an effective strategy for increasing maize yield, improving nitrogen fertilizer use efficiency, and reducing environmental risks.

## 1. Introduction

Intensive nitrogen (N) fertilization has underpinned yield gains in modern cereal-based systems, but it has also contributed to a persistent decoupling between crop N uptake and N inputs [1]. Over the past two decades, excessive agricultural N application has accelerated the leakage of reactive N into water, air, and terrestrial ecosystems through leaching, runoff, and gaseous emissions, thereby aggravating eutrophication, soil acidification, and air pollution [2,3,4]. In China, these environmental costs are increasingly evident, yet synthetic N fertilizer consumption remains high and continues to increase in some regions [5,6]. Reconciling high and stable grain production with increasingly stringent constraints on nitrogen losses is therefore a central challenge for sustainable intensification [7]. This requires not only reducing N rates where possible, but more importantly re-engineering the temporal and spatial patterns of N supply so that they better match crop demand and minimize surplus N in the soil–plant system.

Nitrogen is the primary limiting nutrient in most maize systems, and cereal crops such as maize exhibit high and temporally concentrated N requirements during vegetative growth and the transition to reproductive development [8]. Mineral N fertilizer is the dominant N source in maize production, and historically, increasing N rates has been a straightforward means of increasing yield [9]. Nitrogen availability regulates canopy development, dry mass production, and assimilate partitioning to kernels, thereby strongly influencing final grain yield [10]. Urea is the most widely used N fertilizer globally, accounting for about 70% of total N fertilizer use. However, conventional urea rapidly hydrolyses and dissolves in soil, often releasing N much faster than maize can absorb it. This temporal mismatch means that only part of the applied N is captured by the crop, leading to diminishing yield responses and low nitrogen use efficiency (NUE) [10]. The remaining N is vulnerable to loss via leaching, runoff, and gaseous pathways, thereby increasing the risk of environmental pollution [4,11].

Controlled-release urea (CRU) was developed to partially resolve this mismatch by slowing and extending N release [12]. It is typically produced by coating urea granules with low-water-soluble inorganic or organic polymers, or by embedding N within a porous polymer network, so that N is released more gradually and in closer synchrony with crop growth [13]. The release rate is governed by diffusion through and degradation of the coating material, which can extend N availability into later growth stages. However, the early release from CRU alone may be insufficient to fully satisfy the high N demand during seedling establishment and early vegetative growth. A growing body of work, therefore, suggests that blending CRU with conventional urea may offer a more flexible strategy: readily available N from urea can support early growth, while the CRU fraction maintains a sustained N supply later in the season [14]. Previous studies have shown that optimized N management can simultaneously improve maize yield, NUE, and soil fertility [15], yet the performance of specific CRU–urea blending ratios is often evaluated only in terms of yield or partial NUE indicators, with less emphasis on the underlying N dynamics and environmental implications [16,17].

The black soil region of Northeast China, dominated by fertile Mollisols, is a major maize production base and a strategic reserve for national food security. In recent decades, however, signs of soil quality decline and reduced N availability have emerged, associated with intensive tillage and imbalanced fertilization. In many maize fields, farmers still apply large, one-time doses of readily soluble N fertilizers. This practice tends to generate excessive N supply in early growth stages, inadequate N supply later in the season, low NUE, and substantial N surplus in the soil profile. Although CRU and CRU–urea blends are promising tools for improving N synchrony, quantitative evidence in black soils remains limited regarding how specific blending ratios (i) alter seasonal trajectories of aboveground dry mass and organ-specific N accumulation/remobilization, (ii) reshape the vertical distribution of soil $NO_{3}^{-}$–N and the risk of nitrate movement to deeper layers, and (iii) influence the apparent N balance and economic returns relative to conventional split urea fertilization [16].

Addressing these knowledge gaps is essential for moving from empirical recommendations toward mechanistically informed N management strategies that jointly consider agronomic performance, environmental risk, and farm profitability [1,7]. Therefore, we aimed to (1) quantify whether one-time basal applications of CRU–urea blends reduce soil $NO_{3}^{-}$–N accumulation and apparent N loss relative to split urea management; (2) compare grain yield, dry mass accumulation, N remobilization, and NUE across blended and conventional strategies; and (3) identify an optimal strategy based on integrated agronomic, environmental-risk, and economic indicators for maize production in the black soil region of Northeast China.

## 2. Results

### 2.1. Dynamic Changes in Soil $NO_{3}^{-}$-N Concentration

**Figure 1:**
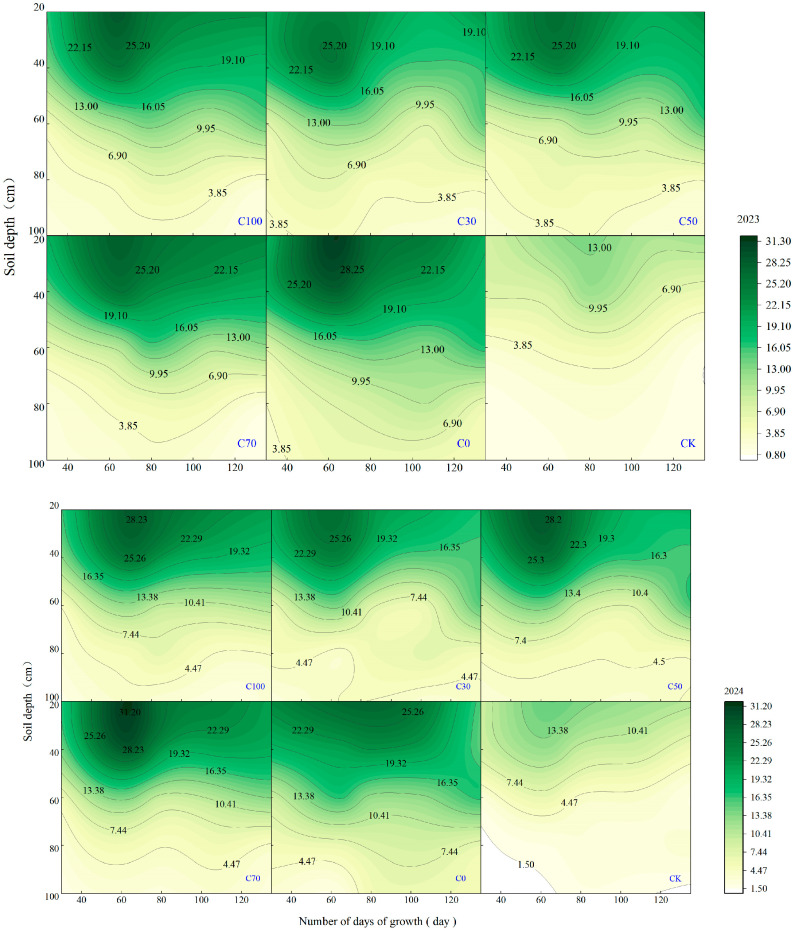
Soil $NO_{3}^{-}$-N concentrations in the 0–100 cm soil profile during V6–R6 under different N treatments in 2023–2024.

Soil $NO_{3}^{-}$-N concentration varied with soil depth, growth stage, and CRU fertilization rate (Figure 1). During the seedling stage, $NO_{3}^{-}$-N was mainly concentrated in the 0–20 cm soil layer, and the concentrations in all fertilization treatments were significantly higher than in CK (*p* < 0.05). By the heading stage, $NO_{3}^{-}$-N had moved downwards in the soil profile, and in the 0–20 cm soil layer, the C0 level of conventional nitrogen fertilization was comparable to that of the high CRU treatment. At maturity, relatively high $NO_{3}^{-}$-N concentrations remained in the 20–40 cm soil layer, comparable to those of the higher CRU treatments. The $NO_{3}^{-}$-N in the 0–20 cm soil layer of C70 was 35.6% higher than C0 in 2023 and 18.2% higher than C0 in 2024. In contrast, in the deeper soil layers (60–100 cm), the $NO_{3}^{-}$-N under the C0 treatment was generally higher, indicating a greater risk of nitrate leaching under conventional urea treatment.

### 2.2. Grain Yield, Protein Yield, and Aboveground Dry Mass

**Figure 2:**
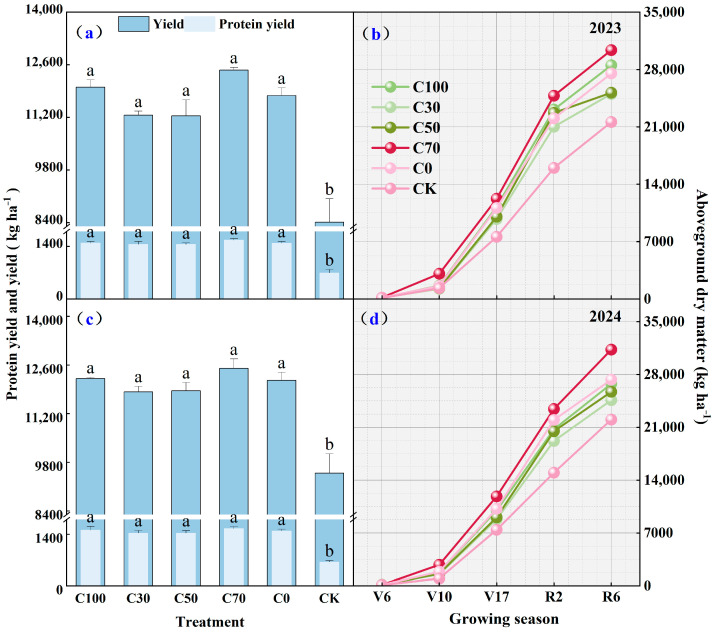
Grain yield, protein yield (**a**,**c**), and aboveground dry matter (**b**,**d**) in 2023 and 2024. Different lowercase letters within the same year indicate significant differences among treatments at *p* < 0.05 (LSD test).

Nitrogen fertilizer treatment significantly affected grain yield and protein yield in both 2023 and 2024 (Figure 2a,c). In 2023, the C70 treatment yielded the highest grain yield, at 12,502.92 kg ha^−1^, higher than the CK, C30, and C50 treatments. The C100 and C0 treatments had intermediate grain yields, not significantly different from the highest yield of C70 (*p* > 0.05). In 2024, the C70 and C0 treatments had the highest grain yields, at 12,457.65 and 11,780.45 kg ha^−1^, respectively, with no significant difference between them (*p* > 0.05). Protein yield showed a similar trend to grain yield, with the C70 treatment having the highest protein yield in both years, reaching 1567.65 and 1551.89 kg ha^−1^, respectively. The dynamics of aboveground dry mass accumulation are shown in Figure 2b,d. Throughout the growing season, the aboveground dry matter mass of all treatments continuously increased. C70 consistently showed the highest aboveground dry matter mass during the reproductive growth stage, reaching 30,373 kg ha^−1^ at R6 in 2023 and 31,309 kg ha^−1^ in 2024. This was 10.3% and 14.5% higher than the final dry matter mass of C0 in 2023 and 2024, respectively. From the V6 to V17 stage, the dry matter accumulation of the C70 treatment was generally higher than that of other CRU treatments.

### 2.3. Nitrogen (N) Accumulation, Remobilization, and N Efficiency

**Figure 3:**
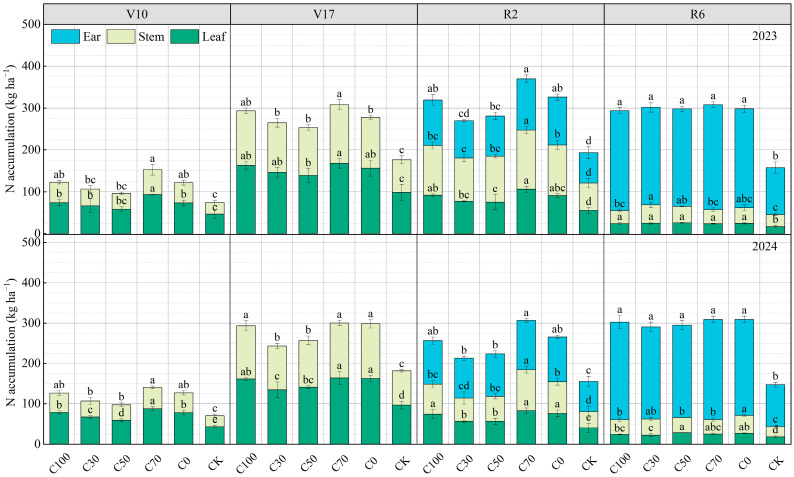
N accumulation in stems, leaves, and ears of maize at V17 and R6 under different N treatments in 2023–2024. Different lowercase letters indicate significant differences among treatments at *p* < 0.05 (LSD test).

Nitrogen accumulation in different plant organs varied markedly among treatments (Figure 3). At the tasseling stage, leaf N accumulation under C70 reached 167.73 and 164.09 kg ha^−1^ in 2023 and 2024, respectively, and these values were significantly higher than those under the conventional N treatment C0 (*p* < 0.05). C100 resulted in leaf N accumulation comparable to that of C0. Stem N showed a similar pattern, with C70 and C0 maintaining significantly higher values than C30, C50, and CK. During the grain-filling stage, ear N accumulation remained highest under C70 and was significantly greater than under C30 and C50. By physiological maturity, differences in ear N among the N-fertilized treatments were not statistically significant, although C70 still showed the numerically highest values of 250.82 and 248.30 kg ha^−1^ in 2023 and 2024, respectively.

**Figure 4:**
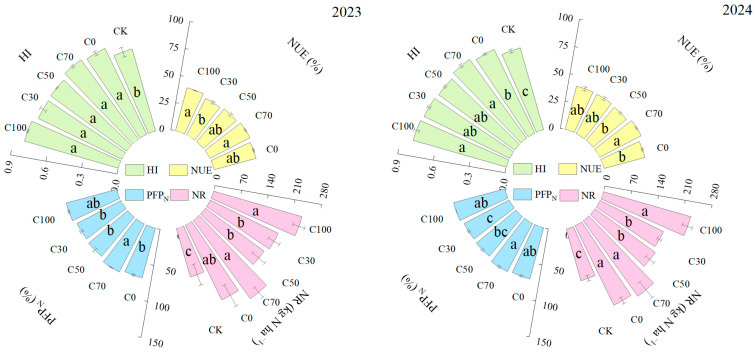
Comparisons of NUE, PFPN, N remobilization (NR), and harvest index (HI) among N treatments in 2023–2024. Different lowercase letters indicate significant differences among treatments at *p* < 0.05 (LSD test).

CRU application also had pronounced effects on N efficiency indices (Figure 4). The conventional N treatment C0 achieved an average NUE of 38.91%. In 2024, C70 resulted in the highest NUE of 42.19%, representing a 10.07% increase relative to C0. PFPN followed a similar pattern, with C70 being 5.95% higher than C0. The amount of N remobilized from vegetative organs to grain was greatest under C70, averaging 245.08 kg ha^−1^ across the two years. This was 16.43% and 5.36% greater than the corresponding values under C0 in 2023 and 2024, respectively. C100 also showed high N remobilization, which did not differ significantly from C70 in 2023 (*p* > 0.05). HI was generally high across all fertilized treatments; however, HI under C70 was 4.35% higher than under C0 in 2024.

### 2.4. Apparent Nitrogen (N) Loss

**Figure 5:**
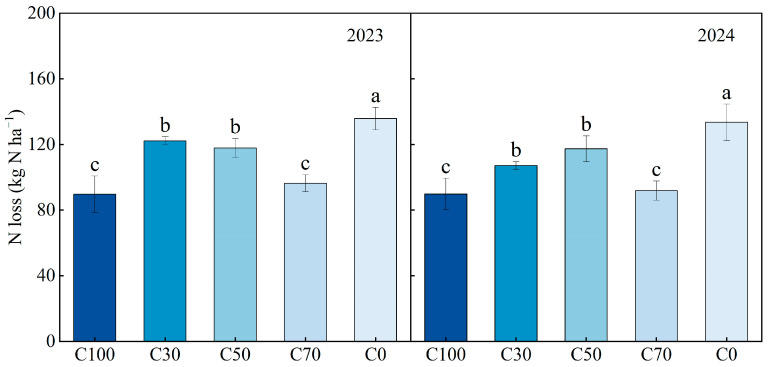
Apparent N loss under different treatments in 2023–2024. Different lowercase letters within the same year indicate significant differences among treatments at *p* < 0.05 (LSD test).

Apparent N loss represents a system-level estimate that integrates multiple loss paths without specifically distinguishing between them (e.g., volatilization, leaching). The difference between the system’s inputs and outputs (crop uptake and soil residue) is assumed to be the loss to the environment. Apparent N loss was significantly influenced by CRU application rate (Figure 5). In both growing seasons, C0 showed the highest apparent N loss, with a two-year mean of 134.64 kg N ha^−1^. Apparent N loss under C70 was 29.04% lower than that under C0 in 2023 and 31.21% lower in 2024. C100 and C70 consistently showed the lowest apparent N losses, and these two treatments did not differ significantly from each other in either year (*p* > 0.05).

### 2.5. Economic Evaluations

Economic returns responded strongly to the CRU application mode (Table 1). C70 resulted in the highest net economic return, averaging US$2817 ha^−1^ across the two years. This was 7.7% and 11.8% greater than the net returns from C0 in 2023 and 2024, respectively. In 2023, the output-to-input ratio was highest under C30, C50, and C70. In 2024, C100 achieved a similarly high output-to-input ratio, whereas C0 consistently exhibited the lowest output-to-input ratio among all fertilized treatments.

### 2.6. Comprehensive Analysis of N Strategies

**Figure 6:**
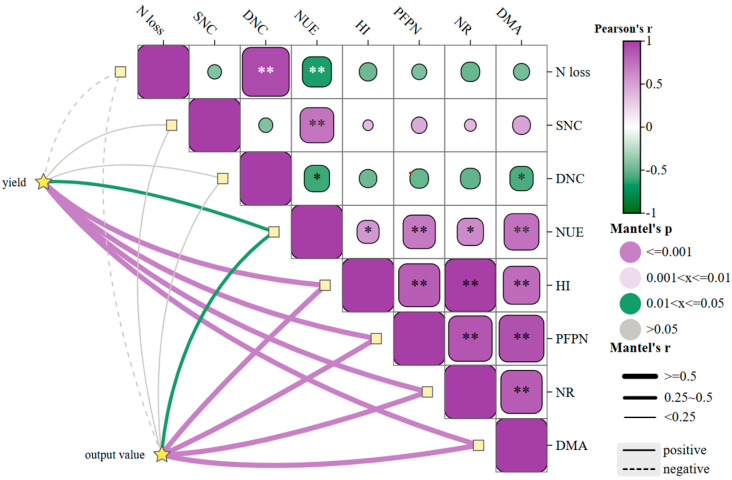
Correlation analysis of key traits. Note: N loss, SNC, DNC, NUE, HI, PFPN, NR, and DMA represent apparent nitrogen loss, nitrate nitrogen concentration in surface soil, nitrate nitrogen concentration in subsoil, nitrogen use efficiency, harvest index, partial productivity, nitrogen transport, and dry matter accumulation, respectively. * indicates *p* < 0.05, and ** indicates *p* < 0.01. The same applies below.

Across all N strategies, the correlation matrix and network (Figure 6) showed a consistent pattern. Yield and output value were strongly and positively correlated with NUE, PFPN, HI, NR, and DMA (*p* < 0.05). Higher yield and output value were always accompanied by higher NUE, PFPN, HI, stronger NR, and greater DMA. In contrast, N loss, SNC, and DNC were negatively correlated with yield, output value, NUE, and PFPN. N loss and DNC showed strong negative correlations with NUE and PFPN. This suggests that DMA and N transport within the plant were closely related to both yield formation and N use efficiency. The correlation pattern shows that, in this system, higher yield and economic return were associated with higher NUE and PFPN and with lower apparent N loss and soil $NO_{3}^{-}$–N (SNC and DNC).

**Figure 7:**
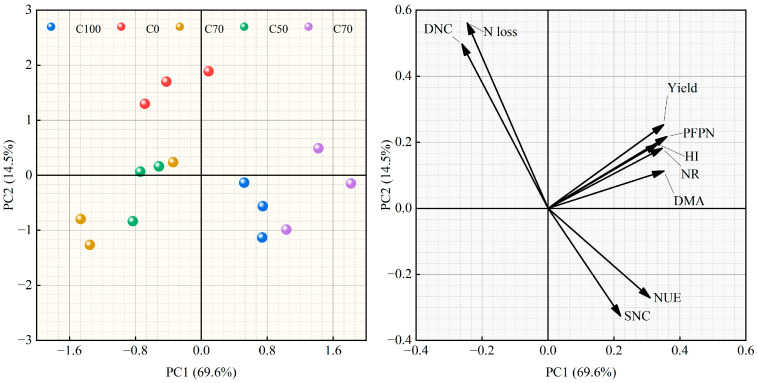
Principal component analysis of yield, economic benefits, N-use efficiency, and N balance under different N strategies.

Principal component analysis of the composite data showed that the first two principal components (PC1 and PC2) together explained 84.1% of the total variance (69.6% and 14.5%, respectively), indicating that most of the differences among the N strategies could be described in a two-dimensional space (Figure 7). In the figure, all N treatments clustered relatively close to the origin, but their positions were clearly arranged along PC1. The traditional split application of urea treatment (C0) was located near the origin, representing the average state of the system, while treatments with higher proportions of CRU (C50 and C70) shifted towards the positive direction of PC1. In contrast, the pure CRU treatment (C100) and the low-CRU mixture treatment (C30) tended to fall on the negative side of PC1. This ordering represents a continuous gradient of overall performance: from less favorable trait combinations under C100 and C30, through the baseline represented by C0, to more favorable combinations under C50 and especially C70.

## 3. Discussion

Intensive nitrogen (N) fertilization in high-yielding maize systems has long been associated with a trade-off between maximizing crop productivity and minimizing environmental N losses [1,2,7]. The present study demonstrates that this trade-off can be partially alleviated by optimizing the spatiotemporal pattern of N supply, rather than by increasing or decreasing the total N input. Specifically, blending controlled-release urea (CRU) with conventional urea at a 70:30 ratio (C70) reshaped N availability in the soil–plant system.

### 3.1. Improved Synchrony Between Nitrogen Supply and Maize Demand

The superior performance of the C70 strategy reflects enhanced synchrony between soil N availability and maize N demand across key growth stages. Conventional split urea application (C0) often produces high concentrations of mineral N early in the growing season, when crop N uptake capacity is limited by low dry mass and shallow root systems, thereby increasing the risk of nitrate loss [10,11]. Conversely, the sole application of CRU (C100) may delay N release excessively, resulting in insufficient N availability during early vegetative growth and constraining canopy development [13].

By combining readily available urea with a larger proportion of CRU, the C70 strategy provided sufficient early-season N to support rapid dry mass accumulation from V6 to V17, while maintaining sustained N availability during tasseling and grain filling [14,17]. Enhanced aboveground dry mass accumulation and higher leaf and stem N accumulation at tasseling under C70 indicate that they improved the role of N supply synchrony in determining reproductive success and kernel set.

### 3.2. Regulation of Nitrate Distribution and Mitigation of Deep N Movement

Beyond temporal synchrony, C70 significantly influenced the vertical distribution of soil nitrate, which is a critical determinant of N loss risk. Under conventional urea management, higher $NO_{3}^{-}$–N concentrations were frequently observed in deeper soil layers (60–100 cm), particularly at later growth stages, indicating a greater tendency for nitrate to move downward beyond the effective rooting zone. Such patterns are commonly associated with increased nitrate leaching risk in temperate agroecosystems [11].

In contrast, C70 retained a greater proportion of $NO_{3}^{-}$–N within the 20–40 cm soil layer at maturity, which coincides with the main zone of active maize root uptake [18]. This suggests that CRU–urea blending can effectively regulate nitrate migration by moderating the rate and timing at which soluble N enters the soil. Similar reductions in deep-soil nitrate accumulation under blended fertilizer strategies have been reported in other maize systems [16,19]. By extending the residence time of inorganic N within the effective root zone, C70 increased the probability of plant N recovery and reduced exposure to leaching pathways.

### 3.3. Enhanced Internal Nitrogen Utilization and Grain Formation

Improved soil N availability under C70 translated into more efficient internal N utilization by maize plants. Higher N remobilization from vegetative organs to grain and a higher harvest index indicate that sustained N availability during reproductive stages supported effective source–sink relationships [8]. Rather than relying solely on late-season soil N uptake, maize plants under C70 were better able to remobilize stored N to meet grain N demand. Maintaining adequate plant N status around tasseling is critical for kernel set, grain filling, and final yield formation [20,21]. The enhanced N remobilization observed under C70 suggests that CRU–urea blending improves not only total N uptake but also the efficiency with which absorbed N is converted into economic yield.

### 3.4. Apparent Nitrogen Loss and System-Level Nitrogen Efficiency

The substantial reduction in apparent N loss under C70—approximately 30% relative to conventional split urea application—highlights the system-level benefits of optimizing N supply patterns. Apparent N loss integrates multiple N loss pathways and reflects the imbalance between N inputs and plant N recovery [2]. Lower apparent N loss under C70 was consistent with reduced deep-soil nitrate accumulation and higher NUE and PFPN [22]. Multivariate analyses further supported these relationships. Principal component analysis positioned C70 in a favorable region characterized by high yield, high NUE, and low apparent N loss, while correlation analysis revealed strong negative relationships between yield and indicators of N surplus and deep nitrate accumulation. Improved N synchrony and redistribution are central mechanisms linking agronomic performance with reduced environmental risk [23].

### 3.5. Economic Performance and Practical Implications

Although CRU has a higher unit cost than conventional urea, the C70 strategy achieved the highest net economic return across both years. Yield gains under C70 more than offset the increased fertilizer cost, and the one-time basal application reduced labor and machinery requirements associated with split fertilization. Similar economic advantages of CRU-based fertilization strategies have been reported in maize systems, where labor availability and management simplicity are key constraints [24].

In summary, the CRU–urea mixed application is a practical approach, and it is particularly important in the black soil region of Northeast China, as it can improve yield, nitrogen fertilizer utilization, and environmental benefits without increasing total nitrogen input. Maintaining high maize yields while minimizing nitrogen loss is crucial for the sustainable development of black soil planting systems [7,18].

## 4. Materials and Methods

### 4.1. Study Site and Materials

**Figure 8:**
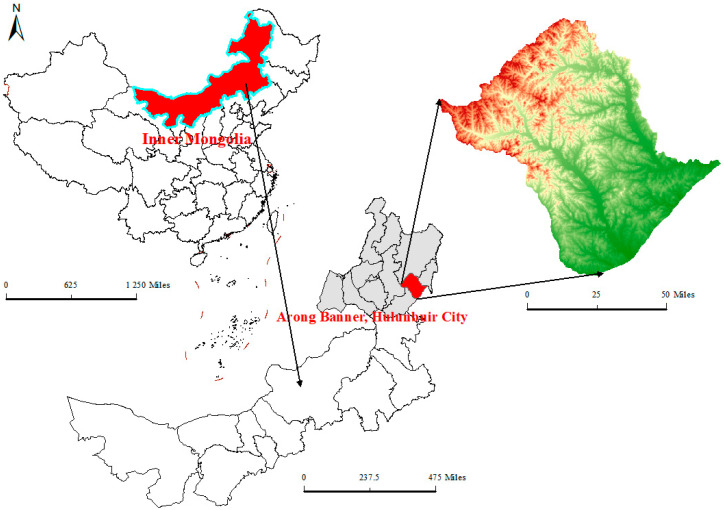
Location of the experimental field at Arong Banner, Hulunbuir, Inner Mongolia, China.

The field experiment was conducted during the 2023 and 2024 growing seasons at the Agricultural Development Center Experimental Base in Arong Banner, Hulunbuir City, Inner Mongolia Autonomous Region, China (48°09′21″ N, 123°28′39″ E; Figure 8). The site is located at an elevation of 232.8 m and has a temperate continental semi-humid climate. The mean annual temperature is 1.7 °C, with ~2800 h of sunshine, an effective accumulated temperature of 2400 °C, annual precipitation of 450 mm, and annual evaporation of 1455 mm. The frost-free period is about 120 days.

The maize Fengyu 8, supplied by the Arong Banner Agricultural Development Center, was used in this study. The fertilizers included urea (46% N), polymer-coated (resin coating) controlled-release urea (Slow-release fertilizer 21-10-11; 40% N, Stanley Fertilizer Co., China; S-shaped release curve with a 90-day release period), diammonium phosphate (18% N, 46% P_2_O_5_), and potassium chloride (60% K_2_O).

**Figure 9:**
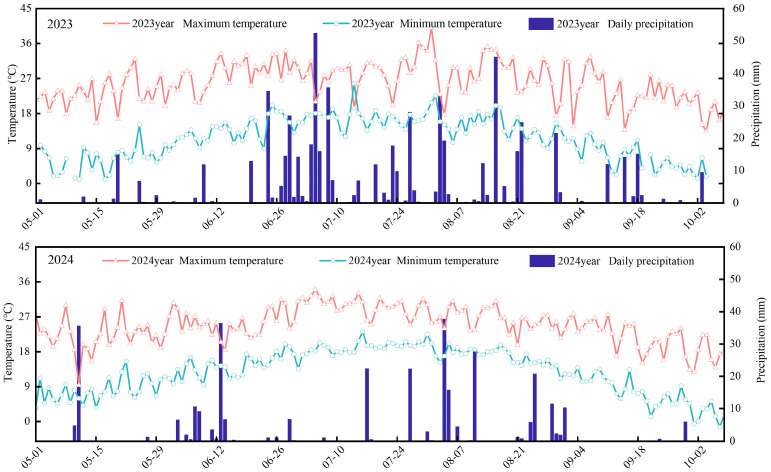
Daily precipitation, maximum air temperature, and minimum air temperature from May to October of 2023 and 2024.

The soil at the experimental site is classified as black soil. The soil properties that were pre-sown in the 0–20 cm layer were: organic matter 24.76 g kg^−1^; total N 2.17 g kg^−1^; alkali-hydrolyzable N 247.2 mg kg^−1^; available P 16.47 mg kg^−1^; available K 247.6 mg kg^−1^; and pH 6.54. The rainfall and temperature dynamics during the growing seasons are shown in Figure 9.

### 4.2. Experimental Design

The experiment followed a randomized complete block design with six N treatments: an unfertilized control (CK); conventional urea applied in splits (40% as a basal application and 20% at each of the jointing, tasseling, and grain-filling stages; C0); and four one-time basal applications of CRU–urea blends in which CRU supplied 100% (C100), 70% (C70), 50% (C50), or 30% (C30) of the total N input, with the remainder supplied by conventional urea. Each treatment was replicated three times, giving a total of 18 plots.

The total N input was the same across all fertilized treatments at 168 kg N ha^−1^. Diammonium phosphate was applied at 187.50 kg ha^−1^, and potassium fertilizer at 142.50 kg ha^−1^, uniformly to all plots. Each plot measured 39 m^2^. Maize was planted on ridges with a row spacing of 65 cm at a target plant density of 75,000 plants ha^−1^. All other agronomic practices followed the local conventional management for high-yielding maize systems. Detailed N application rates for each treatment are presented in Table 2.

### 4.3. Sampling and Analysis

At each sampling time from the V6 (seedling) stage to the R6 (physiological maturity) stage, 3 plants were randomly selected and harvested from each plot, with 3 plots per treatment, for a total of 9 plants. The average value of each plot was used for statistical analysis. At the V17 and R6 stages, the aboveground plant material was separated into stems, leaves, and ears (husk, cob, and grain), and then oven-dried to constant weight. Nitrogen concentration in each maize tissue type was determined using a modified Kjeldahl digestion method. Soil samples were collected from the soil profile and analyzed for $NO_{3}^{-}$-N concentration using an automated flow injection analyzer (FLOWSYS, SYSTEA S.r.l., Anagni (FR), Italy) at the V6–R6 stages.

#### Parameter Calculation

Parameters related to N remobilization, N use efficiency, and apparent N loss were calculated using the following equations:

NR = TN_V17_ − LN_R6_ − SN_R6_ − HN_R6_ − CN_R6_ (1)

(2) $$\mathrm{AEN}=\frac{\mathrm{YN}-\mathrm{YCK}}{N}$$

(3) $$\mathrm{PFPN}=\frac{\mathrm{YN}}{N}$$

Apparent N loss = N_initial_ + AN + N − N_residual_ − N_uptake_ (4)

where NR is N remobilization from vegetative organs to grain between the V17 and R6 stages; TNV17 is total aboveground plant N concentration at the V17 stage; LNR6, SNR6, HNR6, and CNR6 are the N concentration in leaves, stems, husks, and cobs at maturity, respectively; AEN is agronomic N use efficiency; YN is grain yield under a N fertilization treatment; YCK is grain yield in the CK treatment; N is the N application rate; PFPN is the partial factor productivity of applied N; N loss is apparent N loss; N_initial_ is the initial soil $NO_{3}^{-}$-N concentration in the 0–100 cm layer before planting; N_residual_ is the residual soil $NO_{3}^{-}$-N concentration in the 0–100 cm layer at harvest; N_uptake_ is total plant N uptake; and AN is apparent N mineralization, which for the CK treatment was estimated as:

AN = N_uptake_ + N_residual_ − Ni_nitial_ − N

The AN value derived from CK was used to approximate soil N mineralization under fertilized treatments. Apparent N loss represents a system-level estimate and integrates multiple loss pathways without distinguishing among them (e.g., volatilization, leaching).

### 4.4. Economic Analysis

An economic analysis was conducted based on local prices and production costs. Gross return, net return, and the output-to-input ratio were calculated as follows:

Gross return = GY × grain price(5)

Net return = GY × grain price − cost input(6)

(7) $$\mathrm{Output}\text{-}\mathrm{to}\text{-}\mathrm{input}\;\mathrm{ratio}=\frac{\mathrm{Gross}\;\mathrm{return}}{\mathrm{cost}\;\mathrm{input}}$$

where GY is grain yield. The grain price was set at 0.28 US$ kg^−1^, based on surveys of local farmers. The cost input included seeds (148.76 US$ ha^−1^), pesticides (38.24 US$ ha^−1^), machinery (229.46 US$ ha^−1^), and labor for conventional fertilization and topdressing (169.97 US$ ha^−1^), as well as fertilizer costs: urea at 0.37 US$ kg^−1^, controlled-release urea at 0.54 US$ kg^−1^, diammonium phosphate at 0.42 US$ kg^−1^, and potassium fertilizer at 0.17 US$ kg^−1^.

### 4.5. Data Analysis

Microsoft Excel 2025 was used for data organization and preliminary calculations. The effects of N treatments on grain yield, nitrogen use efficiency, and apparent N loss were evaluated by analysis of variance (ANOVA) using SPSS 27.0 (SPSS Inc., Chicago, IL, USA), and data were tested for normality. When treatment effects were significant, means were separated using the least significant difference (LSD) test at *p* < 0.05. All reported values are treatment means based on three replicates. Figures in this paper were produced using Origin 2024 (Origin Lab Corporation, Northampton, MA, USA).

## 5. Conclusions

The strategy of using a 70% CRU mixture with 30% urea (C70) achieves high yields of corn grain and protein, along with efficient nitrogen fertilizer utilization, by improving nitrate retention in the effective root zone and promoting nitrogen remobilization from vegetative organs to grains. This approach also improves economic efficiency and simplifies fertilizer management by reducing the need for multiple topdressings, thereby reducing labor and operational complexity. It provides a balanced strategy for sustaining high maize productivity while improving nitrogen use efficiency and reducing environmental risks. This nitrogen fertilizer management strategy is beneficial for the sustainable and intensive development of maize systems in the black soil region and other temperate continental climate areas with annual precipitation of 450 mm and accumulated temperature of around 2500 °C.

## Figures and Tables

**Table 1:** Economic analysis of different N treatments in 2023 and 2024.

Years	Item	C100	C30	C50	C70	C0	CK
2023	Gross return	3459.68 ± 8.3 ab	3349.20 ± 46.26 b	3360.51 ± 65.39 b	3541.90 ± 77.63 a	3445.46 ± 63.86 ab	2688.98 ± 156.97 c
	Net return	2713.29 ± 8.3 ab	2666.91 ± 46.26 ab	2687.49 ± 65.39 ab	2822.98 ± 77.63 a	2620.66 ± 63.86 b	2112.00 ± 156.97 c
	Output-to-input ratio	4.64 ± 0.01 b	4.91 ± 0.07 a	4.99 ± 0.1 a	4.93 ± 0.11 a	4.18 ± 0.08 c	4.66 ± 0.27 b
2024	Gross return	3401.72 ± 54.18 ab	3153.74 ± 250.6 b	3306.47 ± 156.56 b	3529.08 ± 20.53 a	3337.24 ± 57.9 ab	2287.18 ± 115.64 c
	Net return	2655.34 ± 54.18 ab	2471.45 ± 250.6 b	2633.45 ± 156.56 ab	2810.16 ± 20.53 a	2512.44 ± 57.9 b	1710.20 ± 115.64 c
	Output-to-input ratio	4.56 ± 0.07 a	4.62 ± 0.37 a	4.91 ± 0.23 a	4.91 ± 0.03 a	4.05 ± 0.07 b	3.96 ± 0.2 b

Notes: Mean ± standard deviation. Values followed by different letters within the column are significantly different at *p* < 0.05.

**Table 2:** Proportion of each treatment (fertilizer ratio is clearly labeled as CRU:urea on a N basis).

Treatment	Fertilizer Ratio	Proportion	CRU (kg ha^−1^)	Urea (kg ha^−1^)
C100	CRU	1:0	420.00	—
C30	CRU:Urea	3:7	126.00	255.65
C50	CRU:Urea	5:5	140.00	210.00
C70	CRU:Urea	7:3	294.00	109.57
C0	Urea	0:1	—	365.22
CK	—	0	—	—

## Data Availability

The original contributions presented in this study are included in the article. Further inquiries can be directed to the corresponding author.
