# Correction: Tian et al. CRU–Urea Mixtures Improve Maize Protein Yield and Nitrogen Use Efficiency in the Black Soil Region of Northeast China. *Plants* 2026, *15*, 675

**DOI:** 10.3390/plants15142104

**Published:** 2026-07-08

**Authors:** Lele Tian, Chunyan Yin, Liang Feng, Xiaorong Wu, Li Han, Jinhu Yang, Fang Luo, Ju Zhao, Lijun Li

**Affiliations:** 1 Inner Mongolia Academy of Agricultural & Animal Husbandry Sciences, Huhhot 010031, China; tianlele@emails.imau.edu.cn (L.T.); ycyliuhu@163.com (C.Y.); 13474703792@163.com (L.F.); 2 College of Agronomy, Inner Mongolia Agricultural University, Huhhot 010019, China; shendu2012126636@imau.edu.cn (X.W.); hanli@emails.imau.edu.cn (L.H.); yjh0019@emails.imau.edu.cn (J.Y.); 3 Arong Banner Agricultural Development Center, Hulunbuir 162750, China; 18848177303@163.com

In the original publication [1], there was a mistake in Figure 2 as published. Regarding the significance annotations in Figure 2a,c, the error arose during figure preparation. The grain yield and protein yield data were input in the same spreadsheet, and an incorrect reference was inadvertently assigned when linking the significance label column. As a result, the significance labels for grain yield were mistakenly generated using the label column corresponding to protein yield. This was solely a labeling error caused by an incorrect column reference. The corrected Figure 2 appears below. 

Due to the incorrect significance labels, the description of the 2023 grain yield results was also inaccurately reported. A correction has been made to Section 2.2. Grain Yield, Protein Yield, and Aboveground Dry Mass, Paragraph 1:

In 2023, the C70 treatment had the highest grain yield, at 12,457.65 kg ha^−1^. In 2024, the C70 treatment yielded the highest grain yield, at 12,502.92 kg ha^−1^, higher than the CK, C30, and C50 treatments. The C100 and C0 treatments had intermediate grain yields, not significantly different from the highest yield of C70 (*p* > 0.05).

The authors state that the scientific conclusions are unaffected. This correction was approved by the Academic Editor. The original publication has also been updated.

## Figures and Tables

**Figure 2:**
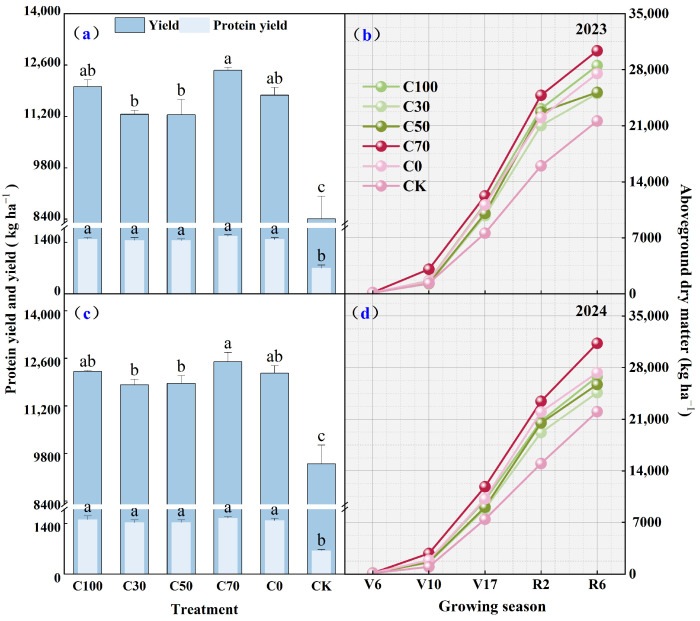
Grain yield, protein yield (**a**,**c**), and aboveground dry matter (**b**,**d**) in 2023 and 2024. Different lowercase letters within the same year indicate significant differences among treatments at *p* < 0.05 (LSD test).
